# Clinical and economic burden of healthcare-associated infections: A prospective cohort study

**DOI:** 10.1371/journal.pone.0282141

**Published:** 2023-02-23

**Authors:** Kidu Gidey, Meles Tekie Gidey, Berhane Yohannes Hailu, Zigbey Brhane Gebreamlak, Yirga Legesse Niriayo

**Affiliations:** 1 Department of Clinical Pharmacy, School of Pharmacy, College of Health Sciences, Mekelle University, Mekelle, Ethiopia; 2 Pharmacoepidemiology and Social Pharmacy Unit, School of Pharmacy, College of Health Sciences, Mekelle University, Mekelle, Ethiopia; 3 Department of Pharmacy, Ayder Comprehensive Specialized Hospital, Mekelle, Ethiopia; Federal University of Acre (UFAC), BRAZIL

## Abstract

**Introduction:**

Healthcare-associated infections (HAIs) have become a serious public health problem. Despite the fact that implementing evidence-based infection control strategies could prevent HAIs and save billions of dollars, Ethiopia lacks national surveillance studies on the rate, economic, and clinical burden of HAIs.

**Objective:**

To assess the clinical and economic burden of HAIs in hospitalized patients at Ayder comprehensive specialized hospital.

**Materials and methods:**

A prospective cohort study design was conducted in patients with and without HAIs. A review of medical records, interviews, and patient bills was used to extract necessary information. The patients in the two arms were matched based on age, sex, Charlson comorbidity index, and ward type. Measurable factors were compared between infected and uninfected patients using the paired ttest or McNemar’s test, as appropriate. Logistic regression was used to identify predictors of in-hospital mortality. Stata 14.1 was used to conduct all analyses.

**Results:**

A total of 408 patients, 204 with HAIs and 204 without HAIs were included in the study. In-hospital mortality was higher in patients with HAI (14.7% vs 7.8%, *P* = 0.028). Patients with HAI stayed an average of 8.3 days longer than controls (18.85 vs 10.59, *P*<0.001). The average direct medical costs for patients with HAI were 3033 Ethiopian birrs (ETB) higher than controls (4826 vs 1793, *P*<0.001). The presence of HAIs (AOR: 2.22, 95% CI: 1.13–4.39) and admission to intensive care units (AOR: 3.39, 95% CI: 1.55–7.40) were significant predictors of in-hospital mortality.

**Conclusion:**

HAIs have a significant impact on in-hospital mortality, the length of extra hospital stays, and extra costs for medical care. Patients admitted to intensive care units and those with HAIs were found to be significant predictors of in-hospital mortality. Interventions must be implemented to prevent HAIs, especially in patients admitted to intensive care units.

## Introduction

Healthcare-associated infections (HAIs) represent a significant burden and safety concern for patients in developing countries. A systematic review of the burden of HAIs in developing countries revealed an overall prevalence of up to 15.5% [1]. Furthermore, in critically ill patients in a developing country, the prevalence of HAIs has been reported to be as high as 34.1% [2].

The prevalence of HAIs in two Ethiopian teaching hospitals was 14.9% [3]. These infections expose patients to potential risks and complications such as resistance of microorganisms to antimicrobials and flora imbalance. This leads to poor long-term clinical outcomes, including prolonged length of hospital stays, increased morbidity, long-term disability, and unnecessary deaths [4, 5] with disproportionate adverse effects in low- and middle-income countries (LMIC) [6].

HAIs not only led to increased morbidity and mortality but also impose a significant financial burden on the health system [7]. The estimated direct annual cost of treating HAIs in the United States ranges from $ 28.4 billion to $ 45 billion, resulting in a heavy burden on the public health system [8]. Such infections are unnecessary adverse events because they can be avoided through proper healthcare worker behavior and adherence to evidence-based infection prevention procedures and guidelines [9].

Implementing comprehensive evidence-based infection control strategies could prevent hundreds of thousands of HAIs and save tens of thousands of lives and billions of dollars [10]. Surveillance studies on the rate, economic burden, and clinical burden of HAIs should be conducted for HAI prevention and control. The availability of published data on the clinical and economic burden of HAIs enhances awareness among health-care providers and authorities. A survey carried out by the World health organization in 2010 showed that only 16% (23 of 147 countries included) of developing countries reported a functioning national surveillance system with some differences between low- and middle-income countries [11]. Although there is a significant body of information on the cost of HAIs from developed countries, translating data from developed countries to situations in developing countries may not be appropriate [12]. Furthermore, financial costs attributable to HAIs are poorly and variably reported in low- and middle-income countries [13].

In Ethiopia, very little attention has been paid to the total burden of HAIs. Although little research has been done to determine the prevalence and risk factors for HAIs, no studies have been conducted on the clinical burden and cost associated with HAIs [3, 14, 15]. The purpose of this study was to assess both the clinical and economic burdens associated with HAIs.

## Materials and methods

### Study setting and period

This study was conducted in the Ayder comprehensive specialized hospital wards. The hospital is one of the leading referrals & teaching hospitals in Ethiopia with a catchment population of about 10 million people. ACSH runs many specialized/non-specialized hospital services, including inpatient and outpatient services. Among the inpatient services, it includes internal medicine, pediatrics, psychiatry, surgery/orthopedics, and gynecology. Patients were recruited from November 2019 to March 2020.

### Study design and population

A prospective cohort study design was conducted. We included all patients with HAI (cases) admitted during the study period. Once enrolled, each patient with HAI (case) was matched in a 1:1 ratio to inpatients without HAIs (controls). The matching criteria were sex, age (±2 years), admission ward, and Charlson comorbidity index. If there were multiple potential controls, the one with the closest admission date to that of a patient with HAI was selected.

We defined the presence of HAI according to the surveillance definition criteria established by the US Centers for Disease Control and Prevention [16]. All hospitalized patients older than 18 years of age with a hospital stay of ≥ 48 hours and admitted to the surgical ward, gynecology ward, medical ward, and intensive care unit of ACSH were included. If a patient without HAIs developed infection within 7 days after selection, he or she was classified as having HAIs. All patients were followed until death or hospital discharge.

### Sample size determination and sampling technique

The sample size was calculated using Statcalc of Epi info version 3.5.1 software package taking into account the following assumptions: 2.8% mortality among controls and 9.7% mortality among cases from a study conducted in Greece [17], 95% CI, a power of 80%, and a case to control ratio of 1:1. This gives a total sample size of 388 (194 cases and 194 controls). Taking a 5% non-response rate, the final sample size was 408 (204 cases and 204 controls). Consecutive patients with HAI were recruited with matched controls to achieve the desired sample size.

### Outcome measures

Our primary outcomes were both clinical and economic outcomes. Clinical outcomes were evaluated in terms of all-cause in-hospital mortality (inpatient mortality) and length of hospital stays. We also determined the predictors of mortality considering the entire study cohort.

Economic outcomes were measured in terms of direct medical costs (total expenditure). The total costs for each patient were estimated using data from the hospital settings (pharmacies, laboratory, microbiologic studies, etc). Patient bills or charges were also used for drugs and other devices purchased from outside of the hospital. Drug costs were estimated based on the average price of the drugs to minimize the effect of price variability on drug costs. All direct costs to the patient including the costs of medications, investigations, imaging, microbiologic studies, hospital bed, procedures, and other relevant costs were included. Indirect costs were not included in the study.

### Data collection

Data were collected using a data collection form (S1 File) which was developed by reviewing different similar studies [18–20]. The data collection form was completed using the patient’s medical record and interviews with the patient. Some information such as socio-demographic characteristics was collected directly from patients or attendants through interviews. All other medical, diagnostics, and treatment characteristics were collected from the patient’s medical record. All patients included in the study were followed daily from admission to discharge. The baseline data were collected at the time of enrollment, and additional data were collected on a daily basis for any additional laboratories, interventions, and patient outcomes.

The following data were recorded for all included patients: demographic characteristics, admission ward, date of admission, type of disease and comorbidities on admission, laboratory investigations, radiology, pharmacologic and non-pharmacologic interventions with their duration, culture and antibiotic susceptibility (when performed for patients with HAIs), date of discharge, hospital outcome, and the cost related to (investigations, radiology, and interventions). We followed the STROBE (Strengthening the Reporting of Observational Studies in Epidemiology) recommendations for reporting cohort studies [21].

Nine health professionals were employed to collect the data. A two-day training was given on the objective and purpose of the study, as well as how to collect the data. The data collection tool was pretested on 20 patients that were excluded from the final analysis.

### Statistical analysis

Data were entered into Epi-Data manager (v.4.2.0.0) and exported to STATA 14.1 (STATA Corporation, Texas, USA) for analysis. Descriptive analysis was performed using mean (standard deviation) for quantitative variables and frequency for categorical variables. The results of the Kolmogorov-Smirnov statistic were used as a test of normality. Continuous variables including length of hospital stay and the hospital cost were compared between the two groups using the paired ttest. Categorical variables were compared using a McNemar’s test. A univariate logistic regression analysis was performed to determine the association of each independent variable with mortality. Subsequently, variables with a p-value<0.25 in the univariate analysis were included in the multivariate logistic regression model to assess the predictive factors of in-hospital mortality. All *P* values were set at less than 0.05 to indicate statistical significance.

### Ethical considerations

This study was approved by the institutional review board (IRB) of Mekelle University, College of Health Sciences (Ref. number: ERC 1225/2019). All the study participants were well informed about the protocol of the study and written informed consent was obtained from all participants. In addition, the confidentiality of personal information was strictly preserved.

## Results

### Socio-demographic and clinical characteristics of the participants

During the study period, a total of 204 patients developed HAIs. A total of 204 matched controls were identified. The mean age (SD) was 42.1 years (17.9) for the cases and 41.9 years (18.8) for the controls. The majority (40.7%) of the patients were from the surgical ward. About 43% of the patients had underlining medical conditions [Table 1].

**Table 1:** Demographic and clinical characteristics of patients with and patients without healthcare-associated infections at ACSH, Nov. 2019-Mar. 2020.

Characteristic	HAIs n (%)	No HAIs (%)	Total n (%)
Age(years)			
Mean age ± SD	42.1 (17.0)	41.1(18.3)	41.6(17.7)
Gender			
Male	101(49.5)	101(49.5)	202(49.5)
Female	103(50.5)	103(50.5)	206(50.5)
Type of ward			
Medical	57(27.9)	57(27.9)	114(27.9)
Gynecology	34(16.7)	34(16.7)	68(16.7)
Surgical	83(40.7)	83(40.7)	166(40.7)
ICU	30(14.7)	30(14.7)	60(14.7)
Comorbidity Charlson index			
Mean ± SD	2.83(2.37)	2.81(2.41)	2.82(2.38)
Underlying medical illness			
No	121(59.3)	112(54.9)	233(57.1)
Yes	83(47.4)	92(45.1)	175(42.9)
Urinary catheter use			
No	102 (50)	138(57.5)	240(58.8)
Yes	102 (50)	66(39.3)	168 (41.2)
Mechanical ventilator use			
No	171 (48.4)	182 (51.6)	353(86.5)
Yes	33 (60)	22 (40)	55(13.5)

HAIs = healthcare-associated infections; ICU = intensive care unit

### Types of healthcare-associated infections

Most of the patients (94%) had only one site of infection. Surgical site infections were the most common type of infection (39.7%), followed by pneumonia and bloodstream infections. A mixed type of infection was also observed in some cases [Table 2].

**Table 2:** Sites of healthcare-associated infections among patients at ACSH, Nov. 2019-Mar. 2020.

Type of infection	No. (%) of patients
One site of infection	192(94.1)
Surgical site infections	81(39.7)
Pneumonia	47(23.0)
Urinary tract infections	21(10.3)
Blood stream infections	37 (18.1)
Gastrointestinal infections	3(1.5)
Skin and soft tissue infections	3 (1.5)
More than one site of infections	12(5.9)

### Outcomes

#### Mortality

Of the total 408 patients, 46 (11.3%) died. The in-hospital mortality was higher in the HAIs group (14.7% vs 7.8%, P-value <0.001) [Table 3].

**Table 3:** Clinical and economic burden of patients with and patients without healthcare-associated infections at ACSH, Nov. 2019-Mar. 2020.

Characteristic	Patients with HAIs n (%)	Patients without HAIs n (%)	Mean difference	P value
In-hospital mortality				
No	174 (85.3)	188(92.2)		
Yes	30(14.7)	16 (7.8)	-	<0.001
Length of hospital stay(days)				
Mean (SD)	18.85(11.6)	10.59 (8.2)	8.3	<0.001
**Costs (Ethiopian birr)**				
Cost of procedures and investigations, mean (SD)	926.1(788.1)	476.5(605.6)	312.9	<0.001
Cost of hospital bed, mean (SD)	572.2(378.1)	327 (262.9)	245.2	<0.001
Cost of drugs, mean (SD)	3346.8(2561.9)	893.1(1088.5)	2453.7	<0.001
Total cost, mean (SD)	4825.9(3059.8)	1792.9(1445.1)	3033.0	<0.001

HAI = hospital acquired infections; SD = standard deviation; ETB = Ethiopian birr

#### Prolongation of hospitalization

The average length of stay in the hospital wards of patients with HAI was 18.85 ± 11.6 days and 10.59 ± 8.2 days for controls: thus, cases stayed on average 8.3 days longer than controls (P<0.001) [Table 3].

#### Economic burden

The average overall costs for patients with HAI were 4826 ETB and 1793 ETB for controls (p value<0.001). Therefore, cases were charged an average of 3033 ETB more than controls [Table 3].

#### Predictors of in-hospital mortality

We performed an association test while controlling for potential confounders to see if there was a significant difference in mortality between those who developed HAIs and those who did not develop HAIs. In the univariable analysis ward admission, catheter use, ventilator use, and the presence of HAIs were significantly associated with in-hospital mortality.

The results of the multivariable logistic regression indicated that patients who were admitted to the ICU ward were more likely to have in-hospital mortality (Adjusted odds ratio (AOR): 3.35, 95% confidence interval (CI): (1.19, 9.48)) than patients who were admitted to the medical ward. Whereas patients admitted to the surgical and gynecology ward were less likely to have mortality compared to patients admitted to the medical ward. Similarly, patients with HAIs were more likely to die (AOR = 2.11, 95% CI: 1.04–4.31) compared to the patients without HAIs [Table 4].

**Table 4:** Univariable and multivariable logistic regression analysis of predictors of in-hospital mortality at ACSH, Northern Ethiopia, 2019.

Characteristic	In-hospital mortality		COR (95%CI)	P value	AOR (95%CI)	P value
	Yes	No				
Age(years)						
Mean age	50.8(15.9)	41.03(18.47)	1.00 (0.98, 1.02)	0.978	0.99 (0.98, 1.02)	0.791
Gender						
Male	20	182	Ref.		Ref.	
Female	26	180	1.31(0.71, 2.44)	0.386	1.92 (0.96, 3.83	0.064
Ward of admission						
Medical	15	99	Ref.		Ref.	
Gynecology	2	66	0.20(0.04, 0.90)	0.036	0.11(0.02, 0.54) *	**0.007**
Surgical	9	157	0.39(0.16, 0.89)	0.027	0.31(0.12, 0.79) *	**0.014**
ICU	20	40	3.30(1.54, 7.08)	0.002	3.35(1.19, 9.48) *	**0.022**
Underlying medical illness						
No	23	210	Ref.		Ref.	
Yes	23	152	0.72(0.39, 1.34)	0.932	0.78(0.38, 1.60)	0.502
Urinary catheter use						
No	15	225	Ref.		Ref.	
Yes	31	137	3.39(1.77, 6.52)	<0.001	2.19 (0.98, 4.88)	0.055
Mechanical ventilator use						
No	34	319	Ref.		Ref.	
Yes	12	43	2.62 (1.26, 5.44)	0.010	2.40(0.88, 6.55)	0.088
HAIs						
No	16	188	Ref.		Ref.	
Yes	30	174	2.03(1.07, 3.85)	0.031	2.11(1.04, 4.31) *	**0.039**

AOR = Adjusted odds ratio; COR = crude odds ratio; HAI = healthcare-associated infections; ICU: intensive care unit; SD = standard deviation

*Indicates significant results in the multivariate regression

## Discussion

This study provides insight and information on the economic and clinical burden of HAIs in different wards of a developing country, Ethiopia. We found that patients with HAI had higher in-hospital mortality, longer hospital stays, and higher costs than patients without HAI.

In-hospital mortality was higher in patients with HAI compared to patients without HAI (14.7% vs 7.8%, P = 0.028). In agreement with our study, a study carried out in a French university hospital revealed that the mortality rate was higher in patients with HAI [20]. This finding may be explained by the fact that patients with HAI had a much higher incidence of organ failure, such as adult respiratory distress syndrome, disseminated intravascular coagulation, acute kidney injury, or shock that could result in patient mortality [22, 23].

HAIs have a significant impact on LOS [24, 25]. In our study, the increase in LOS of HAI was about twice that of uninfected patients, and cases stayed on average 8.3 days longer than controls. Consistent with our study, a study conducted in 68 Chinese hospitals found that the increase in LOS due to HAI in different regions was 8.2 to 12.6 days [26]. Furthermore, the treatment of HAIs requires at least seven days of intravenous antibiotics which could contribute to the prolonged stay.

HAIs were associated with additional costs in the present study. This is supported by numerous other studies conducted in developing and developed countries [12, 18, 27]. This could be due to the need for additional antimicrobials, including very expensive antibiotics. Furthermore, as found in our study, HAIs may result in a longer hospital stay, which may increase the costs associated with the longer hospital stay.

The study also determined predictors of in-hospital mortality among the study cohort. We found that HAIs were significantly associated with in-hospital mortality. This is in agreement with other studies conducted in different settings [10, 28, 29]. ICU patients were also more likely to die than internal medicine patients and other wards. This could be explained by the severity of the illness in intensive care which could contribute to the increase in-hospital mortality.

The findings of this study have important implications. Significant increases in in-hospital mortality, length of stay, and costs require special attention. The prolongation of LOS in patients with HAIs will occupy hospital beds and reduce the number of patients to be admitted. This is a major concern, especially in developing countries where there are few referral hospitals. In addition, the increased cost reported in our study could have been used for preventative measures rather than for the treatment of HAIs. An HAI prevention program will not only pay for itself but will also generate other direct and indirect benefits for patients and society as a whole [30]. The potential savings from HAIs control could be significant in our settings.

HAIs are a major threat to patient safety, but they are highly preventable by promoting various strategies [31]. The availability of hand-washing facilities and waste management materials in the wards is very important [32, 33]. However, studies in Ethiopia have revealed poor hand hygiene practices and medical waste management [14, 34]. Furthermore, antimicrobial stewardship is not available in Ethiopian hospitals. Implementation of all components of antimicrobial stewardship is very important to control infection [35]. Thus, policymakers and healthcare providers should consider implementing antimicrobial stewardship and other infection prevention strategies.

Our study has several limitations. This study was conducted in a single center, therefore, the generalizability of our results to other settings should be approached with caution. The antimicrobial resistance pattern of HAIs was not included due to the small number of patients undergoing culture and sensitivity testing. Our study may not be matched appropriately because we only used four variables for matching. We only reported in-hospital mortality; it would have been preferable to include long-term mortality as well. Another major limitation of this study is the estimation of the length of hospital stay as a time-fixed variable. Patients with HAI typically have long stay in the hospital before becoming infected, and time-invariant methods for estimating excess LOS due to HAI are subject to time-dependent bias that leads to an overestimation of excess LOS. However, we tried to minimize the bias in part by selecting controls whose admission date was closest to that of a patient with HAI. Finally, in this study, we only estimated the direct medical economic burden of HAIs. The costs related to the cost of illness and loss of productivity were not measured in this study which could underestimate the related costs.

We suggest that further research be undertaken to establish the economic burden attributable to HAIs, including all possible costs for more effective HAI surveillance and control. In addition, multicenter studies are needed to confirm these results and we recommend a more in-depth study with large sample size.

## Conclusion

HAIs have a significant impact on in-hospital mortality, the length of extra hospital stays, and extra costs for medical care. Surgical site infections were the most commonly found type of HAIs. Patients admitted to intensive care units and those with HAIs were found to be significant predictors of in-hospital mortality. Interventions must be implemented to prevent HAIs, especially in patients admitted to intensive care units.

## Supporting information

S1 File Questionnaire used to collect information in the study. (DOCX)

S1 Dataset Minimal data set. (DTA)
