# Supplementary material for: Clinical and economic burden of healthcare-associated infections: A prospective cohort study
Source: PLoS One. 2023 Feb 23;18(2):e0282141. doi: 10.1371/journal.pone.0282141 (PMC9949640; doi:10.1371/journal.pone.0282141)
Supplement: S1 File — (DOCX) [file pone.0282141.s001.docx]

**Data collection tool**

- - - 1. **Participants’ socio demographic characteristics and disease related questions**

1. Card number: _____________________
2. Patient’s sex: A. Male  B. Female
3. Age: ________years
   - - 1. **Patient admission ward Information**
4. Admission ward: A. Medical  B. Gynecology  C. obstetric  D. Surgical/Orthopedic  E. ICU
5. Date of admission: _______________________
6. Working diagnosis:
7. What medical risk factor has the patient? Write any comorbid chronic diseases? Or immunosuppressant drugs?
8. Charlson comorbidity index score_______________
9. Has the patient use any medical care device?  Yes  No, if no go to next Q.
10. If yes what is the type of medical care device_______________________________
    - - 1. **Patient infection information after 48 hours admission and above.**

| Has the patient develop infection? |  | Yes |  | No  if no go to next section | |
| --- | --- | --- | --- | --- | --- |
| SSI |  | Superficial infections |  | Under the skin, organs, or implanted material |  |
|  |  |  |  | Bone and Joint |  |
| Pneumonia |  | Ventilator associated |  | None VAP |  |
| UTI |  | With urethral catheter |  | Without indwelling device |  |
| Septicemia |  |  |  |  |  |
| GI. Infection |  |  |  |  |  |
| If other than the list, write here: | | | | |  |
| Date of infection diagnosed: ________________  After how long after admission: ____________________ | | | | |  |

- - - 1. **Laboratory result Data collection**

1. Microbiology laboratory Result
   1. What type of sample collected for Diagnosis? ________________ (Wound swab, Sputum Midstream Urine, Blood, CSF, Stool, Ear swab, Pleural fluid, Pericardial fluid, Ascites fluid, Eye swab, Throat swab, Catheter urine, Urethral swab, Vaginal swap)
2. What type of organism isolated from clinical sample? ___________________________
   - - 1. **Patient discharge or expired information**
          Patient discharged  expired  date _____________________
       2. **Clinical outcome assessment**
3. In hospital mortality  yes  no
4. Average length of hospital stays_____________________________
   - - 1. **Economic burden assessment**

| Drugs medical supply acquisition costs | |  |
| --- | --- | --- |
| **List drugs with Drug name, dose, frequency, duration/ list medical supplies** | **Unit cost** | **Total cost** |
|  |  |  |
|  |  |  |
|  |  |  |
|  |  |  |
|  |  |  |
|  |  |  |
|  |  |  |
|  |  |  |
|  |  |  |
|  |  |  |
| Total antimicrobial drug cost |  |  |
| Total cost of other drugs |  |  |
| **Cost of investigations** | |  |
| **Types of** **investigations** | **Unit price** | **Total** |
| Bacteriology |  |  |
| Hematology |  |  |
| Chemistry |  |  |
| Radiology costs |  |  |
| Cost of procedures |  |  |
| Cost of hospital bed |  |  |
| Other type of costs if available |  |  |
| Overall cost |  |  |
